# The 55K SNP-Based Exploration of QTLs for Spikelet Number Per Spike in a Tetraploid Wheat (*Triticum turgidum* L.) Population: Chinese Landrace “Ailanmai” × Wild Emmer

**DOI:** 10.3389/fpls.2021.732837

**Published:** 2021-08-31

**Authors:** Ziqiang Mo, Jing Zhu, Jiatai Wei, Jieguang Zhou, Qiang Xu, Huaping Tang, Yang Mu, Mei Deng, Qiantao Jiang, Yaxi Liu, Guoyue Chen, Jirui Wang, Pengfei Qi, Wei Li, Yuming Wei, Youliang Zheng, Xiujin Lan, Jian Ma

**Affiliations:** ^1^State Key Laboratory of Crop Gene Exploration and Utilization in Southwest China, Triticeae Research Institute, Sichuan Agricultural University, Chengdu, China; ^2^College of Agronomy, Sichuan Agricultural University, Chengdu, China

**Keywords:** tetraploid wheat, high-quality genetic map, spikelet number per spike, QTL mapping, genetic correlations, potential application

## Abstract

Spikelet number per spike (SNS) is the primary factor that determines wheat yield. Common wheat breeding reduces the genetic diversity among elite germplasm resources, leading to a detrimental effect on future wheat production. It is, therefore, necessary to explore new genetic resources for SNS to increase wheat yield. A tetraploid landrace “Ailanmai” × wild emmer wheat recombinant inbred line (RIL) population was used to construct a genetic map using a wheat 55K single- nucleotide polymorphism (SNP) array. The linkage map containing 1,150 bin markers with a total genetic distance of 2,411.8 cm was obtained. Based on the phenotypic data from the eight environments and best linear unbiased prediction (BLUP) values, five quantitative trait loci (QTLs) for SNS were identified, explaining 6.71–29.40% of the phenotypic variation. Two of them, *QSns.sau-AM-2B.2* and *QSns.sau-AM-3B.2*, were detected as a major and novel QTL. Their effects were further validated in two additional F_2_ populations using tightly linked kompetitive allele-specific PCR (KASP) markers. Potential candidate genes within the physical intervals of the corresponding QTLs were predicted to participate in inflorescence development and spikelet formation. Genetic associations between SNS and other agronomic traits were also detected and analyzed. This study demonstrates the feasibility of the wheat 55K SNP array developed for common wheat in the genetic mapping of tetraploid population and shows the potential application of wheat-related species in wheat improvement programs.

## Introduction

Wheat (*Triticum* sp.) is among the most widely planted crops globally that provides about 20% of the calories consumed by humans (http://www.fao.org/faostat). Breeding of high-yield wheat cultivars is a sustainable approach to meet the demands of a growing human population. Wheat yield is determined by spike number per unit area (SNPA), kernel number per spike (KNS), and kernel weight (KW). KNS is jointly determined by the spikelet number per spike (SNS) and the kernel number per spikelet (KNL). SNS depends on the number of lateral spikelets produced before the spike meristem transitions to the terminal spikelet. It is a complex quantitative trait greatly affected by genetic and environmental factors (Gao et al., 2019; Kuzay et al., 2019). Therefore, dissecting the genetic mechanism of SNS at the quantitative trait locus (QTL) level can provide more insights into the role of SNS in yield formation (Ma et al., 2019a; Yao et al., 2019; Chen et al., 2020; Li et al., 2021).

A few genes related to spikelet number and morphology have been reported. For example, *WHEAT FRIZZY PANICLE* (*WFZP*) gene encodes a member of the APETALA2/ethylene response transcription factor (AP2/ERF) family, which influences the supernumerary spikelet trait in common wheat (Dobrovolskaya et al., 2015). The regulatory expression of the two pivotal flowering genes, *Photoperiod-1* (*Ppd-1*) and *FLOWERING LOCUS T* (*FT*), increases the number of fertile spikelets (Boden et al., 2015). The inflorescence growth rate and the development of paired spikelets are controlled by the *TEOSINTE BRANCHED1* (*TB1*) expression level (Dixon et al., 2018). Similarly, the ortholog of the rice *APO1, WHEAT ORTHOLOG OF APO1* (*WAPO1*), affects the spikelet number (Kuzay et al., 2019). The overexpression of *TaTFL-2D* increases the number of spikelets and florets (Wang et al., 2017).

More loci for SNS should thus be identified and utilized owing to its important role in yield formation. Nonetheless, common wheat breeding reduces the genetic diversity among elite germplasm resources, thus adversely affecting wheat yield (Cavanagh et al., 2013; Sthapit et al., 2020). Notably, numerous genetic resources for agronomic traits and disease resistance from wheat-related species may effectively solve future wheat production challenges (Zaïm et al., 2017; El Haddad et al., 2021).

Wild emmer wheat (*Triticum dicoccoides*) is a self-pollinated plant with brittle spikes and elongated kernels. It has two sets of homologous chromosomes (BBAA), resulting most likely from the chromosome reduplication after the natural hybridization by *Aegilops speltoides* (BB) and *Triticum urartu* (AA) (Özkan et al., 2011). As the progenitor of modern cultivated tetraploid and hexaploid wheat, wild emmer wheat is the secondary gene pool of common wheat (Peng et al., 2003). It has a rich genetic diversity for multi-disease resistances and important agronomic traits, thereby playing a potentially significant role in wheat breeding (Xie and Nevo, 2008; Yu et al., 2020). For example, *Yr15* derived from wild emmer wheat confers resistance to yellow rust (Klymiuk et al., 2018). The allele *GNI-A1* with a reduced function increases the number of fertile florets per spikelet (Sakuma et al., 2019). In addition, it is estimated that only about 10–20% of the wild alleles have been used in modern wheat cultivars (Peleg et al., 2008). In cognizance of this, exploiting the genetic resources of wild emmer wheat is crucial in solving the existing bottleneck in wheat breeding. Wheat landraces are also the primary materials for wheat improvement. They contain unique gene resources preserved under long-term natural selection and human intervention with strong adaptability to local environmental conditions and high production potential (Lopes et al., 2015; Crossa et al., 2016).

In the current research, a high-quality genetic map was constructed based on a recombinant inbred line (RIL) population developed from the cross between a Chinese landrace “Ailanmai” and wild emmer using the wheat 55K single nucleotide polymorphism (SNP) array to identify QTLs for SNS, and major and novel QTLs were validated in different genetic backgrounds by kompetitive allele-specific PCR (KASP) markers.

## Materials and Methods

### Plant Materials

**Figure 1:**
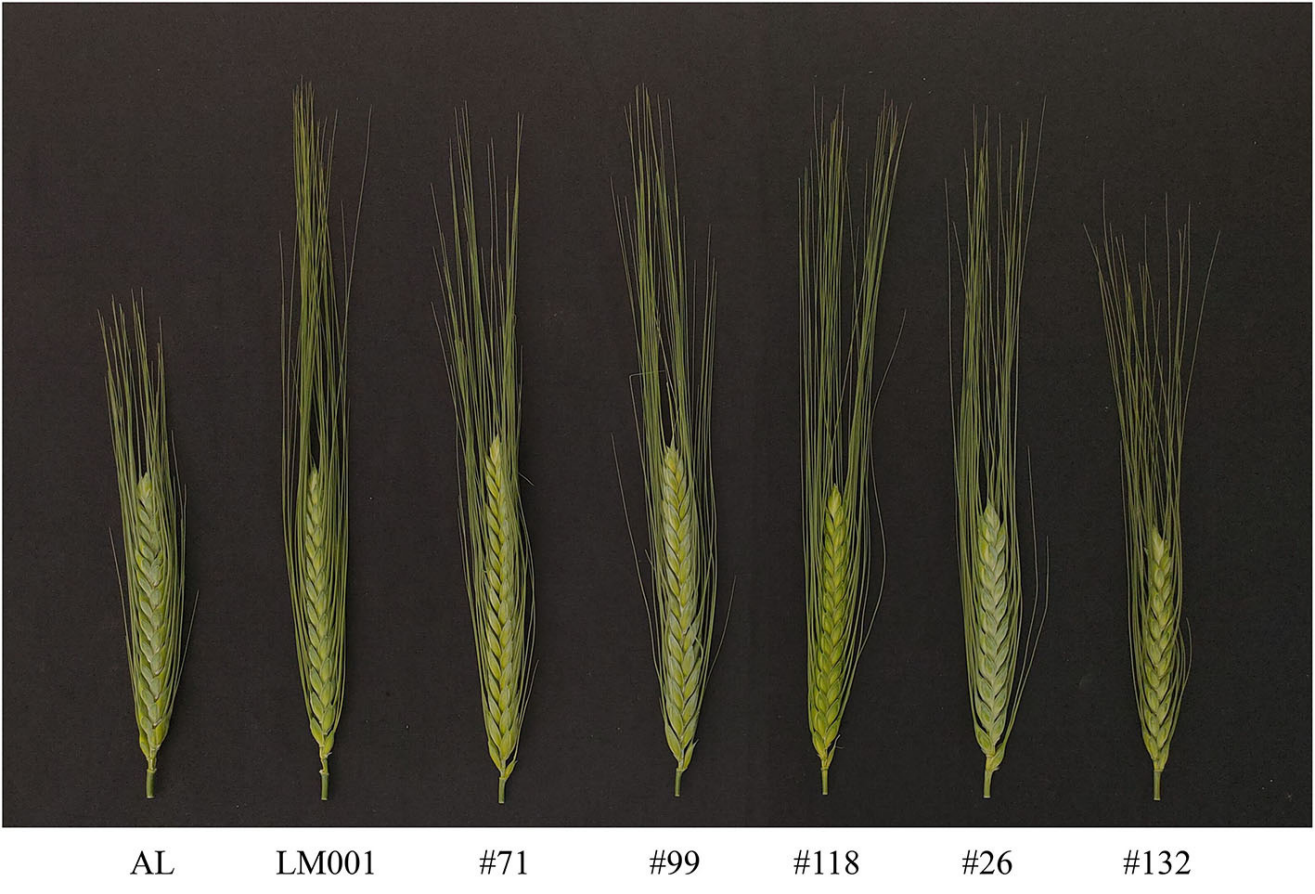
The spike morphology of the parents and representative lines from the AM population (scale bar = 2 cm).

A tetraploid wheat population (AM population) containing 121 F_8_ RILs (including two parental lines), derived from a Chinese landrace “Ailanmai” (AL, *Triticum turgidum* L. cv. Ailanmai, 2n = 28, AABB) and a wild emmer accession (LM001, *T*. *turgidum* subsp. *dicoccoides*, 2n = 28, AABB) was used in this study. AL is a durum wheat landrace native to the Sichuan Province, China. It has the advantages of dwarf, multiple floret, and strong environmental adaptability (Liu et al., 1999). LM001 has the characteristics of long awns, non-free threshability, and less kernels per spikelet. AM RIL population was developed to aim at identifying favorable alleles from wheat-related species to speed up their breeding utilization. The spike morphology of the parents and representative lines are shown in Figure 1. Major and novel SNS QTLs identified in the AM population were validated in the two populations, including LM001 **×** PI193877 (*T. turgidum* ssp. *dicoccon*) (139 F_2_ lines) and AL **×** AS2268 (*Triticum carthlicum* Nevski) (100 F_2_ lines). The abovementioned plant materials were preserved by Triticeae Research Institute, Sichuan Agricultural University, Sichuan, China.

### Trait Measurement and Statistical Analysis

The trait measurements of AM population were performed in the eight environments: Chongzhou (103°38′ E, 30°32′ N) in 2017, 2018, 2019, 2020, and 2021 (E_1_, E_2_, E_3_, E_4_, and E_7_), Wenjiang (103°51′ E, 30°43′ N) in 2020 and 2021 (E_5_ and E_8_), and Ya'an (103°0′ E, 29°58′ N) in 2020 (E_6_). Field experiments adopted a randomized complete block design and were managed according to the conventional practices of wheat production. The plot for a single line was space-planted (0.1 m) in a single 1.5 m row with a 0.3 m of row spacing. The agronomic traits were measured using the methods described by Liu et al. (2020a). SNS was measured by counting the spikelet number of the main -spike per plant; plant height (PH) was determined by the distance from the soil surface to the tip of the main spike (excluding awns) per plant; spike length (SL) was measured by the length from the base to the tip of the main spike (excluding awns) per plant; KNS was counted as the kernel number of the main spike per plant; spike density (SD) was calculated by dividing the SNS by SL; 1,000 kernel weight (TKW) was calculated as 10 times the average weight of 100 seeds with three repetitions in each line. Anthesis date (AD) was the number of days between sowing and half of the plants flowering in each line (Ma et al., 2019a). Spike extension length (SEL) was measured as the distance between the base of the main spike and the petiole of the flag leaf per plant (Li et al., 2020). A minimum of four disease-free plants with consistent growth status were selected for measurements. The environmental information of the surveyed agronomic traits of AM population is listed in Supplementary Table 1. Two F_2_ validation populations were grown at Chongzhou in 2021, and five disease-free plants were selected to measure SNS.

The IBM SPSS Statistics 25 (Armonk, NY, USA) was used to analyze the phenotypic data, including Pearson's correlation, frequency distribution, SE, and the Student's *t*-test (*p* < 0.05). The broad-sense heritability (*H*^2^) and best linear unbiased prediction (BLUP) of the agronomic traits from multiple environments were calculated using SAS version 9.1 (Cary, NC, USA).

### Construction of the Genetic Linkage Map

High-quality genomic DNA was isolated from fresh leaves using the Plant Genomic DNA Kit (Tiangen Biotech, Beijing, China). The DNA of 119 RILs and 2 parental lines was subsequently genotyped by CapitalBio Technology (Beijing, China) using the wheat 55K SNP array, and RILs were used for linkage map construction.

A genetic map was constructed according to the methods described by Liu et al. (2018). Firstly, the poly high-resolution markers from A and B genomes showing a minor allele frequency (≥0.3) among the mapping populations were retained for a subsequent analysis. Secondly, the BIN function of QTL IciMapping 4.2 (Meng et al., 2015) was used to analyze the remaining markers based on their segregation patterns in the mapping population, with the parameters “distortion value” and “missing rate” being set as 0.01 and 20%, respectively. A single marker with the lowest “missing rate” in each bin (bin marker) was selected to construct genetic maps. Finally, the bin markers were grouped and sorted using the Kosambi mapping function in IciMapping 4.2 with the logarithm of odds (LOD) ≥6 after the preliminary analysis of SNP markers with the LOD values ranging from 2 to 10. The genetic maps were drawn using MapChart 2.3.2 (Voorrips, 2002). The syntenic relationships between the genetic and physical maps of the bin markers were further presented using the Strawberry Perl 5.24.0.1.

### QTL Analysis

Quantitative trait loci analysis was performed using the inclusive composite interval mapping in the biparental population (BIP) module (mapping method: ICIM-ADD) of QTLs IciMapping (Li et al., 2007a). The LOD threshold was set as 3.4 based on a test of 1,000 permutations in IciMapping, which was used to determine the threshold corresponding to a genome-wide error rate of 5% (*p* < 0.05). In addition, QTLs detected in only a single environment were removed. The epistatic interaction of SNS QTLs was also identified using the BIP module (mapping method: ICIM-EPI) of IciMapping (Step = 5 cm, PIN = 0.0001, and LOD ≥ 5.0) (Li et al., 2008). The QTLs-by-environment interactions (QEIs) of the SNS trait were analyzed using the function of multi-environment trial module (mapping method: ICIM-ADD) in IciMapping (Step = 1 cm, PIN = 0.001, and LOD ≥ 7.0) (Li et al., 2015).

Based on the initial QTL mapping results, we developed new KASP markers to densify a genetic map and narrow the mapping interval. Specifically, the genomic DNA of two parental lines was genotyped using the wheat 660K SNP array from CapitalBio Technology (Beijing, China). Polymorphic SNPs detected in the initial mapping region were converted to KASP markers to genotype the mapping population, which was integrated into a genetic map for QTL remapping.

Quantitative trait loci identified in at least five environments (including BLUP) and explained more than 10% of the phenotypic variation were regarded as the major ones, while those having the common flanking markers were treated as a single one. QTLs were then named according to the Genetic Nomenclature provision (http://wheat.pw.usda.gov/ggpages/wgc/98/Intro.htm). In the naming system, “Sau” represented “Sichuan Agricultural University.” The name of RILs “AM” was also added to the name of QTLs to distinguish them from the others reported earlier.

Sequences of the markers were blasted against the durum wheat (Svevo) (Maccaferri et al., 2019), wild emmer (Zavitan; v2.0) (Zhu et al., 2019), and common wheat genotype “Chinese Spring” (CS; v2.1) (Zhu et al., 2021) genome to identify their physical positions. Svevo and Zavitan genomes were used when considering the physical positions of QTLs detected from AL and LM001, respectively. The predicted genes mapped between the flanking markers and their functional annotations and expression patterns were obtained from the Triticeae Multi-omics Center (http://202.194.139.32/).

### QTL Validation

The tightly linked SNP markers of major and novel SNS QTLs were used to develop KASP markers. KASP primers were designed and applied according to the method of Tan et al. (2017). Each validation population was divided into two haplotype groups (with homozygous alleles from any parent) according to the presence of their alleles in parents and progenies. Phenotypic differences between the two groups were analyzed using Student's *t*-test (*p* < 0.05).

## Results

### SNP Markers and Genetic Map

As suggested by the Affymetrix Company, the high-probability probes from the poly high-resolution group only were reserved. Thus, 8,724 SNPs with minor allele frequency (≥0.3) among the mapping populations were retained for the following analysis. Subsequently, 823 markers from the D genome were rejected. Finally, 7,901 SNP markers were classified into 1,192 bins, and a single marker with the lowest “missing rate” in each bin (bin marker) was screened to construct a genetic map. The linkage analysis results revealed that 1,150 bin markers were mapped to 15 linkage groups. One linkage group was constructed for each chromosome except chromosome 3B, which had two (Table 1 and Supplementary Table 2). Based on the bin information, 6,537 mapped markers (including 1,150 bin markers and 5,387 cosegregated markers) were integrated into a genetic map with a total length of 2,411.8 cm (Table 1 and Supplementary Table 3). The length of a genetic map in each linkage group was in the range of between 49.7 (3B1) and 225.1 cm (5A). The average distance of bin markers was in the range of between 1.09 (4B) and 3.64 cm (3B2), with an average density of one bin marker per 2.10 cm. The number of mapped markers was in the range of between 37 on the chromosome 3B1 and 944 on the chromosome 2A (Table 1 and Supplementary Table 3). Notably, 56% and 44% of the mapped markers were distributed on A and B subgenomes, respectively.

**Table 1:** Information of single-nucleotide polymorphism (SNP) markers in the constructed genetic map.

**Chromosome**	**Group**	**Number of bin markers**	**Number of mapped markers**	**Length** **(cM)**	**Density (cM/bin)**
1A	1	101	586	153.3	1.52
1B	1	69	488	149.3	2.16
2A	1	66	944	161.8	2.45
2B	1	92	424	183.7	2.00
3A	1	99	517	161.0	1.63
3B	1	17	37	49.7	2.92
	2	27	174	98.4	3.64
4A	1	80	282	199.9	2.50
4B	1	77	422	83.7	1.09
5A	1	92	298	225.1	2.45
5B	1	121	674	207.5	1.72
6A	1	60	636	167.0	2.78
6B	1	58	160	162.5	2.80
7A	1	92	395	214.2	2.33
7B	1	99	500	194.8	1.97
A genome	7	590	3,658	1282.2	2.17
B genome	8	560	2,879	1129.6	2.02
Total	15	1,150	6,537	2411.8	2.10

### Comparison of Genetic and Physical Maps

**Figure 2:**
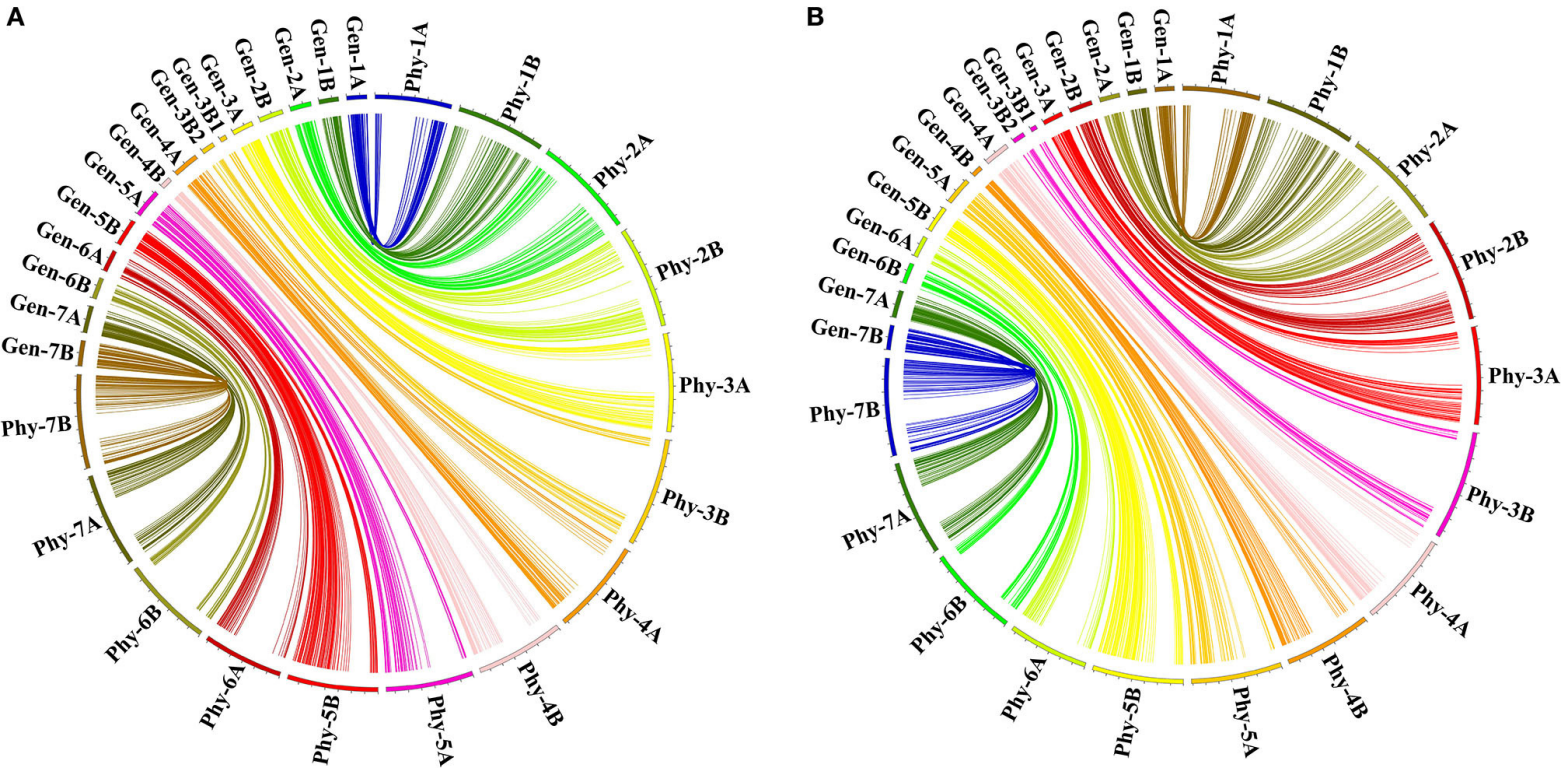
The syntenic relationships between the genetic and physical maps of bin markers. Gen-1A to Gen-7B represented the 15 chromosomal genetic maps used in the current study; Phy-1A to Phy-7B represented the 14 chromosomal physical maps of **(A)** durum wheat and **(B)** wild emmer genomes.

Considering that the parent accessions AL and LM001 were durum and wild emmer wheat, respectively, the sequences of the 6,537 mapped markers were blasted against durum wheat and wild emmer genomes to identify their physical positions (Supplementary Table 3). The genetic chromosomal locations of the 1,150 bin markers were compared with their physical chromosomal locations in the durum wheat genome. Among them, 1,087 (94.52%) markers showed good consistencies (Figure 2A), and 63 (5.48%) markers had inconsistent chromosomal locations. In the wild emmer genome, the genetic chromosomal locations of 71 (6.17%) markers were inconsistent with physical chromosomal locations, whereas the remaining markers (1,079, 93.83%) were well-matched (Figure 2B). Notably, 1,026 (89.22%) bin markers had consistent chromosomal locations in a genetic map, the durum wheat genome, and the wild emmer genome. Similarly, the genetic maps of the 6,537 mapped markers were compared with their physical maps (Supplementary Table 3). 6,242 (95.49%) and 6,249 (95.59%) markers showed consistent chromosomal locations in the durum wheat and wild emmer genomes, respectively, whereas 6,031 (92.26%) markers had consistent chromosomal locations in a genetic map and two physical maps.

### Phenotypic Variation of SNS and Correlations Between SNS and Other Agronomic Traits

Spikelet number per spike of AL and LM001 in multiple environments was in the range of between 25.00 and 27.00, and 23.33 and 26.00, respectively (Table 2). SNS of AM RILs was in the range of between 15.33 and 34.00. There were no significant differences in SNS between the two parental lines. However, a continuous variation and bidirectional transgressive segregation were observed in the RIL population. These results suggested that it was feasible to analyze the SNS loci in the current population.

**Table 2:** Phenotypic data and heritability (*H*^2^) of spikelet number per spike (SNS) for the AM population in multiple environments.

**Environment**	**Parents**		**AL** **×** **LM001 (AM)**			
	**AL**	**LM001**	**Min–Max**	**Mean**	**SD**	***H*^**2**^**
E_1_	25.00	24.33	20.00–31.67	25.96	2.56	
E_2_	26.00	23.33	15.33–33.00	24.41	3.22	
E_3_	26.33	26.00	17.00–31.25	24.81	2.80	
E_4_	26.00	25.40	18.00–32.00	26.21	2.87	
E_5_	27.00	24.60	18.50–31.00	24.99	2.42	
E_6_	25.80	25.40	20.00–34.00	26.76	2.69	
E_7_	23.40	24.25	15.75–31.00	23.68	2.62	
E_8_	25.44	24.67	18.50–30.67	24.93	2.55	
BLUP	25.60	24.78	18.41–30.10	25.22	2.12	0.85

*BLUP phenotype values based on the best linear unbiased prediction; SD, standard deviation; H^2^, the broad-sense heritability*.

**Figure 3:**
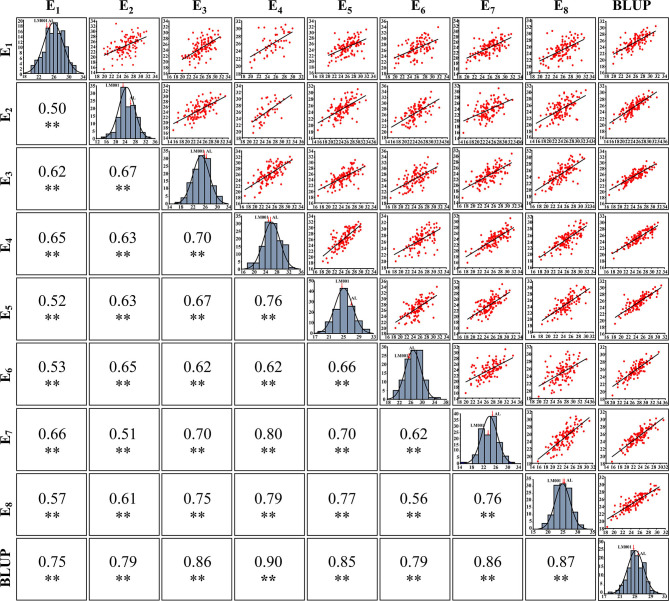
The phenotype, frequency distribution, and correlation coefficients of spikelet number per spike (SNS) in multiple environments. **Significance level at *p* < 0.01.

The broad-sense *H*^2^ of SNS was 0.85 across all environments (Table 2), indicating that the SNS trait was mainly controlled by genetic factors. A strong interaction was detected between SNS and environments, with significant correlation coefficients ranging between 0.50 and 0.90 (Figure 3). The frequency distribution of SNS in multiple environments indicated that it was polygenically inherited (Figure 3).

Pearson's correlations were analyzed between SNS and seven other agronomic traits based on the BLUP values (Table 3). SNS was significantly and positively correlated with PH, AD, SL, SEL, KNS, and SD. However, SNS was not significantly correlated with TKW.

**Table 3:** Correlations between SNS and other agronomic traits in the AM population.

**Trait**	**Spikelet number per spike (SNS)**
Plant height (PH)	0.30^**^
Anthesis date (AD)	0.34^**^
Spike length (SL)	0.70^**^
Spike extension length (SEL)	0.23^*^
Kernel number per spike (KNS)	0.36^**^
Spike density (SD)	0.32^**^
Thousand kernel weight (TKW)	0.14

* *Significance level at p < 0.05*.

** *Significance level at p < 0.01*.

### QTL Mapping of SNS in Single-Environment Analysis

Five QTLs for SNS were identified and distributed on chromosomes 5A (1 QTL), 2B (2), and 3B (2), with LOD scores ranging between 3.55 and 21.49 (Table 4). They explained 6.71–29.40% of the phenotypic variation. *QSns.sau-AM-5A, QSns.sau-AM-2B.2*, and *QSns.sau-AM-3B.2* were detected in at least five environments and explained more than 10% of the phenotypic variation, which were treated as major QTLs. And the other two QTLs were identified as minor QTLs.

**Table 4:** Quantitative trait loci (QTLs) for SNS identified in multiple environments in the AM population.

**QTL**	**Env**.	**Interval (cM)**	**Left marker**	**Right marker**	**LOD**	**PVE (%)**	**Add**	**Physical position (Mb)**
*QSns.sau-AM-5A* *QSns.sau-AM-2B.1* *QSns.sau-AM-2B.2* *QSns.sau-AM-3B.1* *QSns.sau-AM-3B.2*	E_1_ E_3_ E_4_ E_7_ BLUP E_5_ E_8_ E_1_ E_2_ E_3_ E_4_ E_5_ E_7_ E_8_ BLUP E_4_ E_6_ E_8_ E_1_ E_2_ E_3_ E_7_ BLUP	116.1–131.9 131.9–135.2 116.1–131.9 116.1–131.9 116.1–131.9 113.7–114.6 113.7–114.6 131.3–132.2 131.3–132.2 131.3–132.2 131.3–132.2 130.0–131.3 130.0–131.3 131.3–132.2 131.3–132.2 22.7–42.5 22.7–42.5 22.7–42.5 48.3–49.7 47.0–48.3 48.3–49.7 47.0–48.3 48.3–49.7	AX-108790581 AX-111499991 AX-108790581 AX-108790581 AX-108790581 AX-109421413 AX-109421413 KASP-AX-109422178 KASP-AX-109422178 KASP-AX-109422178 KASP-AX-109422178 KASP-AX-95072014 KASP-AX-95072014 KASP-AX-109422178 KASP-AX-109422178 AX-110399975 AX-110399975 AX-110399975 AX-109393346 AX-109876826 AX-109393346 AX-109876826 AX-109393346	AX-111499991 AX-111596791 AX-111499991 AX-111499991 AX-111499991 AX-109506426 AX-109506426 AX-111588559 AX-111588559 AX-111588559 AX-111588559 KASP-AX-109422178 KASP-AX-109422178 AX-111588559 AX-111588559 AX-109050064 AX-109050064 AX-109050064 AX-110548993 AX-109393346 AX-110548993 AX-109393346 AX-110548993	5.62 4.01 3.55 8.17 6.61 3.90 3.82 9.02 5.10 11.14 7.35 4.97 21.49 8.87 15.97 6.61 3.97 7.63 4.82 3.86 5.84 14.77 8.64	12.07 8.27 10.34 9.18 12.09 15.66 7.54 17.99 9.09 26.57 15.79 20.39 25.60 19.32 29.40 17.54 11.85 16.78 9.04 6.71 12.67 15.46 14.27	−0.95 −0.83 −0.95 −0.85 −0.75 −0.75 −0.63 −1.16 −1.06 −1.48 −1.17 −0.85 −1.41 −1.00 −1.16 1.26 1.02 0.95 0.85 0.94 1.05 1.12 0.83	557.72–571.41^a^ 710.11–717.87^a^ 731.25–731.60^a^ 25.95–47.17^b^ 65.87–72.59^b^

*Env., environment; BLUP, best linear unbiased prediction; LOD, logarithm of odds; PVE, phenotype variance explained; Add, additive effect*.

*Positive values: positive alleles from AL, negative values: positive alleles from LM001. Physical position^a^: the flanking marker sequences of QTLs which positive alleles detected from LM001 were blasted against wild emmer genome to get physical positions; Physical position^b^: those detected from AL were blasted against durum wheat genome to get physical positions*.

**Figure 4:**
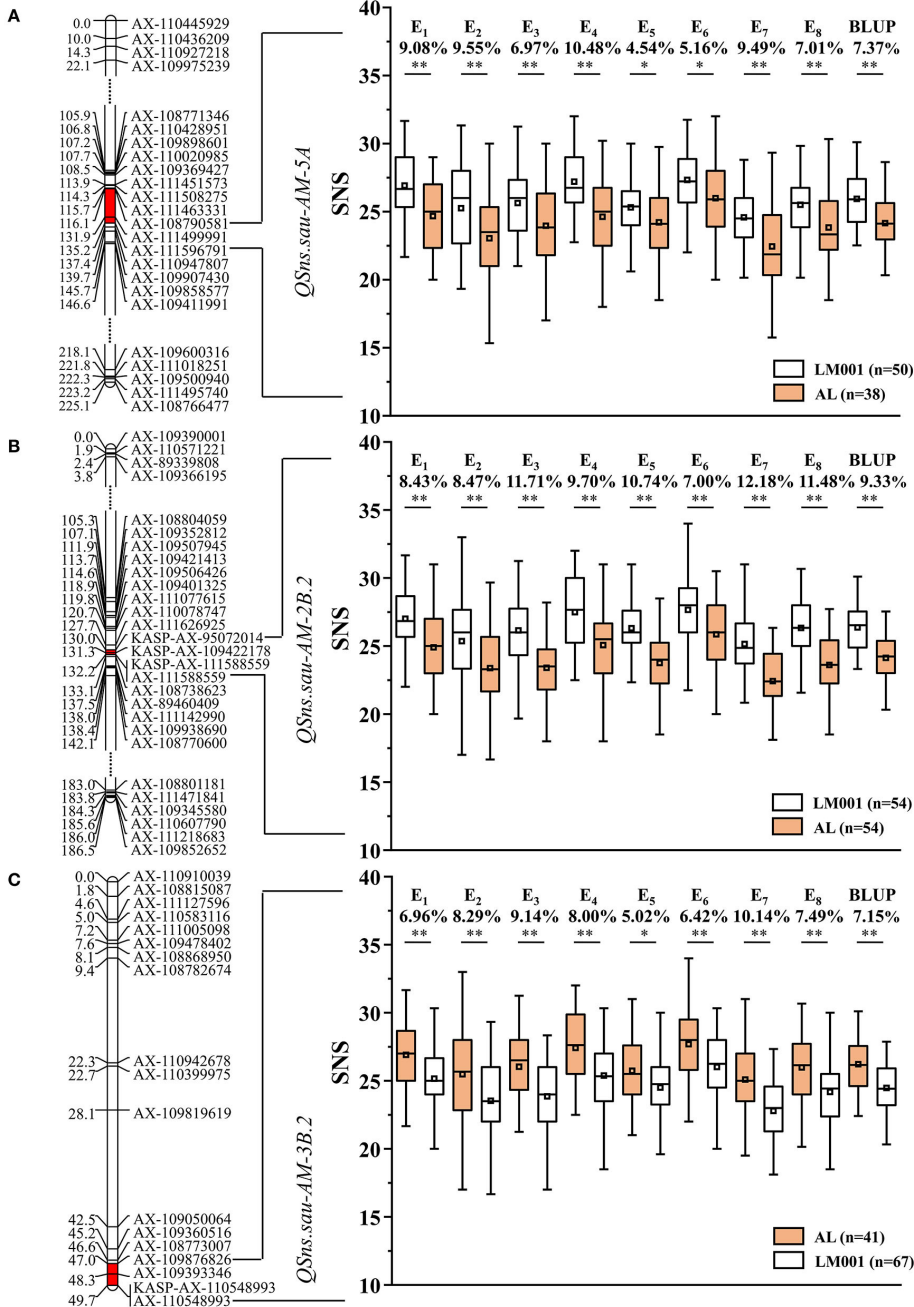
AM recombinant inbred lines (RILs) were divided into two haplotype groups based on the genotype of flanking markers, and the SNS differences caused by the corresponding quantitative trait loci (QTL) were represented. Genetic maps of **(A)** *QSns.sau-AM-5A*, **(B)** *QSns.sau-AM-2B.2*, and **(C)** *QSns.sau-AM-3B.2* and their effects. *Significance level at *p* < 0.05, **Significance level at *p* < 0.01.

*QSns.sau-AM-5A* was detected in five environments and located in a 19.1-cM region between AX-108790581 and AX-111596791. It accounted for 8.27–12.09% of the phenotypic variation (Table 4). AM population was divided into two haplotype groups based on the genotype of the flanking markers of QTLs, and the positive effect of *QSns.sau-AY-5A.2* increased SNS significantly by 4.54–10.48% (Figure 4A).

*QSns.sau-AM-2B.2* was stably identified in all environments, explaining 9.09–29.40% of the phenotypic variation (Table 4). It was firstly mapped between AX-111626925 and AX-111588559 (Figure 4B). AX-95072014 and AX-109422178 were polymorphic SNPs from the wheat 660K SNP array detected in the initial mapping interval. They were converted to KASP markers (KASP-AX-95072014 and KASP-AX-109422178) to genotype the mapping population, which were integrated into a genetic map for QTL remapping. The remapping results showed that *QSns.sau-AM-2B.2* was located in a 2.2-cM region between KASP-AX-95072014 and AX-111588559. The additive effect of *QSns.sau-AM-2B.2* was negative, indicating that an increase in SNS allele was contributed by LM001. The positive effect of *QSns.sau-AM-2B.2* on increasing SNS in a single environment was in the range from 7.00% to 12.18% (Figure 4B).

*QSns.sau-AM-3B.2* was detected in five environments (Table 4). It can explain up to 15.46% of the phenotypic variation. The positive allele of *QSns.sau-AM-3B.2* was from AL, and RILs containing the favorable allele of *QSns.sau-AM-3B*.2 had a higher SNS than those containing a negative one. The positive effect of *QSns.sau-AM-3B.2* significantly increased SNS by 5.02–10.14% in a single environment (Figure 4C).

### Epistatic Interaction and Multi-environment Analysis of SNS QTL

Two pairs of epistatic QTLs were detected for a SNS trait (Supplementary Table 4). Their LOD and PVE values were 5.19 and 5.92, and 15.26% and 32.49%, respectively. All interactions were identified in a single environment, indicating that an epistatic interaction had less consistency across multiple environments. Furthermore, there were no epistatic interactions between five QTL for SNS detected in a single-environment analysis (Table 4 and Supplementary Table 4).

Multi-environment QTL mapping was performed to identify the significant QTLs across multiple environments and QEI effects (Supplementary Table 5). Totally, 17 putative QTLs for SNS were detected using the QEI analysis: one each on chromosomes 1A, 2A, 3A, 4A, and 7A, two each on chromosomes 5A, 6A, and 3B, and three on chromosomes 2B and 5B. Among them, 12 QTLs exhibited to be environment-specific, while the other 5 QTLs were regarded as significant ones in all the 8 environments (*p* < 0.05). For the QTL stability, the absolute value of additive average effects of *QSns.sau-AM-5A, QSns.sau-AM-2B.1, QSns.sau-AM-2B.2*, and *QSns.sau-AM-3B.2* were larger than interaction effects, further inferring that they were stably expressed in multiple environments. *QSns.sau-AM-2B.2* had the largest LOD = 65.29 and LOD (A) = 59.87, and had the highest average additive effect (−1.07), suggesting that the corresponding allele from AL would reduce SNS by 1.07 compared to the population average.

*QSns.sau-AM-3B.2* had the largest LOD (A by E) = 9.04, which had strong QEI. In addition, the mapping interval estimated by the single-environment analysis and QEI analysis fluctuates slightly for some QTLs (Table 4 and Supplementary Table 5). A comprehensive consideration of the two mapping models was more conducive to develop tightly linked markers for marker-assisted breeding and predict the potential candidate genes of major QTLs.

### Effects of Major QTLs on Increasing SNS

**Figure 5:**
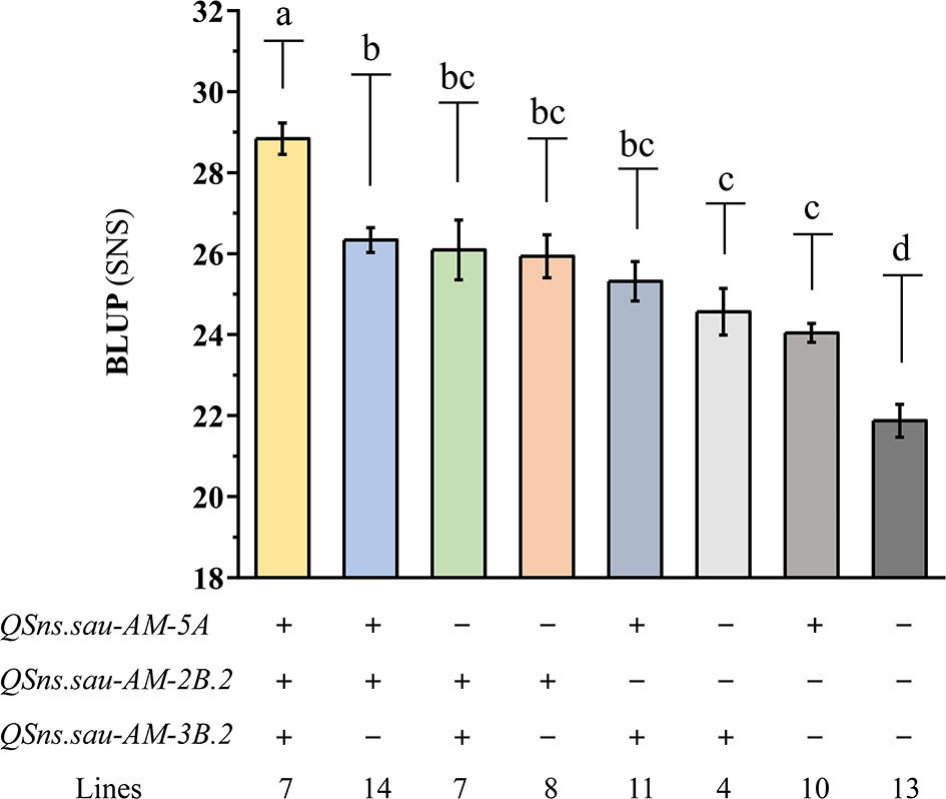
The additive effects of *QSns.sau-AM-5A, QSns.sau-AM-2B.2*, and *QSns.sau-AM-3B.2* on increasing SNS. “+” and “–” represented haplotype group with and without the positive allele of the corresponding QTL based on the genotype of flanking markers, respectively. Letters a, b, c, and d represent a significant difference among haplotype groups.

The effects of three QTLs (*QSns.sau-AM-5A, QSns.sau-AM-2B.2*, and *QSns.sau-AM-3B.2*) on increasing the SNS were further revealed in the mapping population based on the genotypes of the flanking markers (Figure 5). Compared to the haplotype group that carried the negative alleles of three QTLs, haplotype groups with the favorable alleles from at least one QTL significantly increased SNS. The haplotype group containing the favorable alleles from three QTLs showed a significant increase in SNS than the group containing the favorable alleles from two QTLs or one QTL. No significant differences were detected among the three haplotype groups with any two positive alleles. *QSns.sau-AM-2B.2* had the largest individual positive effect on SNS. However, there was no significant difference between the individual effect of three QTLs on increasing SNS.

### Validation of Major and Novel SNS QTLs

**Figure 6:**
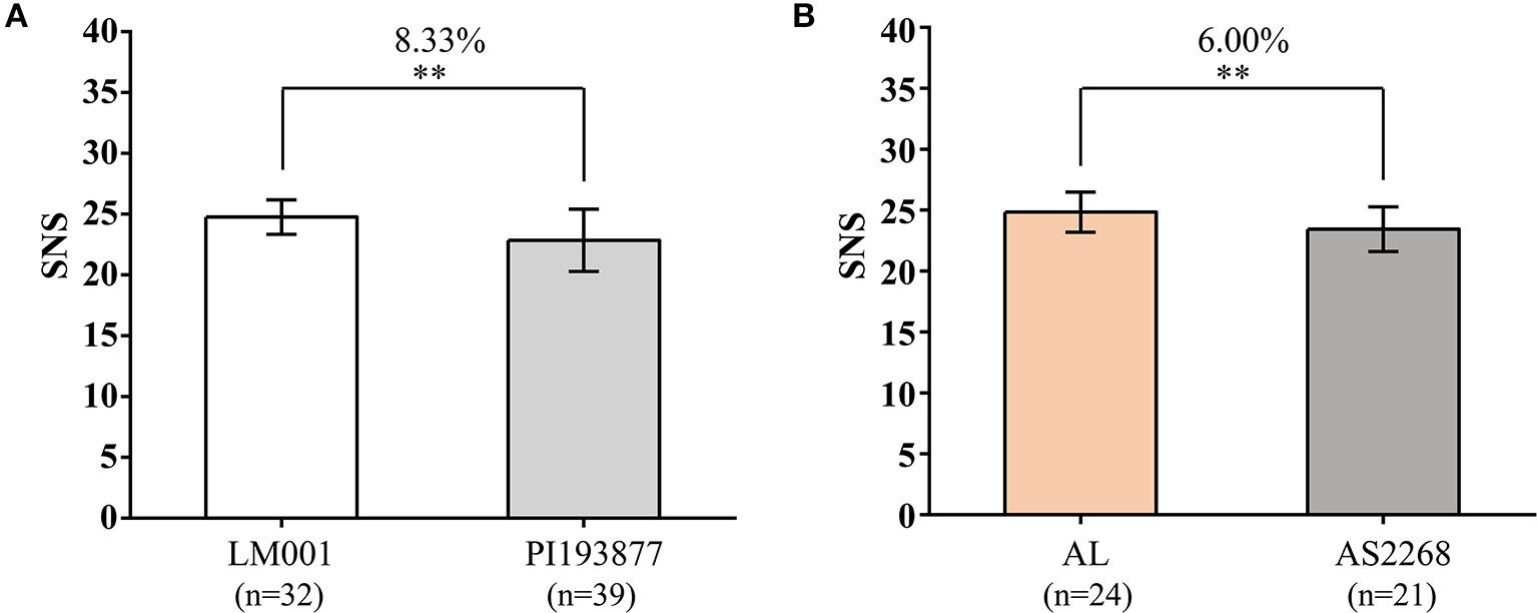
Validation of **(A)** *QSns.sau-AM-2B.2* (KASP-AX-111588559) and **(B)** *QSns.sau-AM-3B.2* (KASP-AX-110548993) in LM001 × PI193877 and AL × AS2268 populations by Student's *t*-test, respectively. **Significance level at *p* < 0.01.

The SNP markers closely linked to *QSns.sau-AM-2B.2* (AX-111588559) and *QSns.sau-AM-3B.2* (AX-110548993) were converted into KASP markers, to verify their effects in the two additional populations (Supplementary Table 6 and Figure 6). Because the favorable alleles of *QSns.sau-AM-2B.2* and *QSns.sau-AM-3B.2* were from LM001 and AL, respectively, haplotype group with homozygous alleles of LM001 showed a significant increase in SNS by 8.33% (Figure 6A) and those from AL increased SNS significantly by 6.00% (Figure 6B).

## Discussion

### Exploration of the SNS Loci From Tetraploid Wheat Species Using the Wheat 55K SNP Array

Previous studies have reported on SNS in tetraploid wheat at the QTL level. Peng et al. (2001) identified four SNS QTLs, explaining a total of 49.3% of the phenotypic variation in a wild emmer × durum wheat F_2_ population genotyped using restriction fragment length polymorphism (RFLP) markers. Genotyping of the durum × cultivated emmer wheat RIL population with the 9K iSelect SNP chip identified four SNS QTL on chromosomes 1B, 3B, 7A, and 7B (Faris et al., 2014a). In the same time, a linkage map of the chromosome 2A generated in the Langdon × Langdon-wild emmer wheat substitution line population consisting of 23 simple sequence repeat (SSR) markers was used to identify an SNS QTL, *QSpn.fcu-2A*, explaining 19.4% of a phenotypic variation (Faris et al., 2014b). SNS QTL, *QSpn.fcu-1B*, and *QSpn.fcu-5A* have been identified in a durum × cultivated emmer wheat population genotyped using the 90K iSelect array (Sharma et al., 2019). These reports enhance our understanding of the genetic mechanism of SNS in tetraploid wheat.

The wheat 55K SNP array was developed based on the selection and optimization of the 660K SNP array, which was designed based on hexaploid, tetraploid, and diploid wheat, and possessed the advantages of being efficient and genome-specific (Sun et al., 2020). It has been widely used for genotyping and genetic loci identification of agronomic traits and disease resistance in bread wheat (Ren et al., 2018; Ma et al., 2019b, 2020; Liu et al., 2020b; Tu et al., 2021). To the knowledge of the author, this is the first study to adopt the wheat 55K SNP array to construct the genetic linkage map of a tetraploid wheat RIL population. Our results confirm the feasibility of using a 55K SNP array developed for common wheat for the genetic mapping of tetraploid wheat, thus proving its broad utilization.

### Comparison of the Major SNS QTLs Identified With Those of Previous Studies

There was no significant difference for SNS detected between the parental lines in multiple environments (Table 2). The phenotypic data of SNS in the eight environments showed approximately a normal distribution (Figure 4). Three major and stably expressed QTLs for SNS were identified. This phenomenon is attributed to the transgressive inheritance in progenies that result in the bidirectional segregation of SNS. Several genes controlling SNS may inhibit each other in a given parental genotype resulting in the absence of the corresponding phenotype in the parents. However, a single locus produced by genetic recombination between parental genotypes can display a conspicuous phenotype in the progenies without interference from the other loci that control the same trait. The major SNS loci in the AM RIL population can thus be identified. This is not uncommon in the past studies of QTL identification based on RIL populations (Liu et al., 2020a; Shang et al., 2020).

Physical locations of the known SNS QTLs were detected to verify whether major QTLs were novel (Supplementary Table 7). In the wild emmer genome, *QSns.sau-AM-5A* was physically located on a chromosome arm 5AL at 557.72–571.41 Mb, overlapping with *QSps.ccsu-5A.2* (568.96–662.44 Mb) (Kumar et al., 2007) and *QTL1755_5A* (569.50–579.91 Mb) (Peng et al., 2003), indicating that they are likely alleles. *QSns.sau-AM-2B.2* was located at 731.25–731.60 Mb on a chromosome arm 2BL in the wild emmer genome. Previous studies have detected SNS QTLs on a chromosome arm 2BS. However, none of them has been reported on 2BL. For example, *Qspn-2007* was located on a chromosome arm 2BS with the closest marker Xgwm429 (Manickavelu et al., 2011). *QFsn.sdau-2B-1* was located on a chromosome arm 2BS, explaining up to 28.43% of the phenotypic variation (Deng et al., 2017). Some genes regulating SNS, such as *Ppd-B1* (Mohler et al., 2004) and *WFZP-2B* (Dobrovolskaya et al., 2015), have also been detected on a chromosome arm 2BS. In the durum wheat genome, *QSns.sau-AM-3B.2* was located on chromosome arm 3BS at 65.87–72.59 Mb. Similarly, Cui et al. (2012) identified two QTLs, *QSpn.WJ.3B.1* and *QSpn.WY.3B.1*, all closely linked to the locus Xgwm566. Azadi et al. (2015) detected three QTLs for SNS on a chromosome arm 3BL. Comparing the physical positions of the three major QTLs with those previously reported indicates that *QSns.sau-AY-2B.2* and *QSns.sau-AY-3B.2* are probably the novel loci controlling SNS in tetraploid wheat.

*QSns.sau-AY-2B.2* and *QSns.sau-AY-3B.2* were also considered stable QTLs by QEI analysis (Supplementary Table 5). In addition, their effects on increasing SNS were validated in other genetic backgrounds using tightly linked KASP markers (Figure 6). This fully demonstrates the application potential of *QSns.sau-AY-2B.2* and *QSns.sau-AY-3B.2* in marker-assisted breeding in widely changing environments. Stable QTLs across environments will lead to improved genotype-to-phenotype prediction, thereby increasing the genetic gain in breeding programs (Alimi et al., 2013; Li et al., 2015; Yu et al., 2016).

### Potential Candidate Genes in the Physical Intervals of *QSns.sau-AM-2B.2*

*QSns.sau-AM-2B.2* was physical located in a 0.35-Mb region of the wild emmer genome, and four genes and three functional orthologs were annotated (Supplementary Table 8A). *QSns.sau-AM-3B.2* was located in a 6.72-Mb interval of durum wheat genome with 62 genes and 38 functional orthologs (Supplementary Table 8B). Because the physical interval of *QSns.sau-AM-2B.2* contains only three orthologs, it is valuable to analyze function annotations and expression patterns to predict candidate genes.

*TRIDC2BG075290* encodes a protein kinase, which involves in cell signal transduction and plant growth. Previous research reported that the wheat protein kinase gene *TaSnRK2.9*-5A was significantly correlated with high TKW and high kernel per spike (Ur Rehman et al., 2019), suggesting that protein kinase can have a positive impact on agronomic traits. In addition, *Stomatal opening factor 1* (*OST1* and *SnRK2.6*) is a homologous gene of *TRIDC2BG075290* in *Arabidopsis*. *OST1* can promote the expression of *FLOWERING LOCUS C* (*FLC*) to participate in the regulation of flowering by ABA (Wang et al., 2013), which may affect inflorescence development and the formation of spikelets. *TRIDC2BG075310* and *TRIDC2BG075320* both encode SAUR-like auxin-responsive protein. It has been reported that the plant SAUR-like auxin-responsive protein family is involved in the growth of roots, hypocotyls, leaves, and stamens (Chae et al., 2012; Hou et al., 2013). Expression pattern analyses showed that *TRIDC2BG075290* has higher expression levels in spikes and flag leaf (Supplementary Figure 1). *TRIDC2BG075310* has a high expression level throughout the spike development stage, while *TRIDC2BG075320* is only expressed in spikes. Therefore, we speculate that these three functional orthologs may affect the wheat inflorescence development, which can be further determined by fine mapping and gene cloning in future.

### Phenotypic Correlations of Agronomic Traits

Spikelet number per spike was significantly and positively correlated with PH in this study (Table 3). This finding was consistent with that of Ajmal et al. (2009) and Wu et al. (2012). We speculate that SL is part of PH, and the taller plants have a long spike with more spikelets (Yao et al., 2011). There was a significant and positive relationship between SNS and AD (Table 3), consistent with the results of Ma et al. (2019a). It is supposed that wheat plants that take a longer time to flower tend to produce longer spikes with more spikelets (Shaw et al., 2013; Kamran et al., 2014; Boden et al., 2015). These results indicate that AD plays an important role in spikelet formation.

Similarly, there was a strong significant and positive correlation between SNS and SL (Table 3). The increase in SL requires a longer spike development stage combined with optimal temperature and light to promote spikelet germination (Shaw et al., 2013). As a whole, floret fertility determines the KNS in wheat. More spikelets produce more fertile florets, thereby increasing the KNS (Gao et al., 2019; Sakuma et al., 2019). This reason potentially caused SNS to be significantly and positively correlated with KNS (Table 3). Moreover, SNS was significantly and positively correlated with SD because SD is the ratio of SNS to SL (Table 3).

It is postulated that there is a negative correlation between SNS and TKW (Li et al., 2007b; Ma et al., 2019a). The negative correlation is attributed to source limitations during kernel filling or more kernels with lower KW in the distal positions of a spike (Quintero et al., 2018). Previous studies also suggest that SNS is negatively correlated with SEL because of competition in product assimilation between SNS and SEL (Bancal, 2008; Li et al., 2020). However, these conclusions are inconsistent with the findings of this study. Herein, SNS was positively correlated with TKW and SEL (Table 3). It probably indicates that the loci controlling SNS play an individual role without interacting with SEL. Previous studies postulate that SEL has a special function in photosynthesis and nutrients and water storage (Bridgemohan and Bridgemohan, 2014; Ávila-Lovera et al., 2017). Therefore, a longer SEL is more conducive for the ventilation and transportation of more nutrients and water to kernels, thereby increasing the KW.

These conclusions are vital to a comprehensive understanding of the complex relationships in agronomic traits, and provide new insights into wheat yield improvement. KNS is one of the primary determinants of grain yield. Therefore, wheat breeding programs should focus on increasing SNS under moderate SD in this study.

## Conclusion

Herein, the wheat 55K SNP array was successfully used to construct a high-quality genetic map that identified five QTLs for SNS in a tetraploid wheat RIL population. The additive effects of the major SNS QTLs in increasing SNS were revealed in the mapping population. *QSns.sau-AM-2B.2* and *QSns.sau-AM-3B.2* were major and novel QTLs, and their effects were successfully validated in other genetic backgrounds using closely linked KASP markers. The genetic correlations between SNS and other agronomic traits were also evaluated. The novel genetic loci of SNS and tightly linked KASP markers will be valuable in marker-assisted breeding and further gene cloning.

## Data Availability Statement

The datasets presented in this study can be found in online repositories. The names of the repository/repositories and accession number(s) can be found in the article/Supplementary Materials.

## Author Contributions

ZM and JZhu conducted the study and drafted this manuscript. JWe and JZho participated in phenotype measurement and data analysis. QX and HT helped to conduct in field work and data analysis. YM, MD, QJ, and YL participated in data collection and analysis. GC, JWa, PQ, and WL did QTL analysis and manuscript revision. YW and YZ discussed the results and revised the manuscript. XL guided the study and revised the manuscript. JM designed the experiments, guided the entire study, participated in data analysis, and wrote and extensively revised this manuscript. All authors participated in the research and approved the final manuscript.

## Conflict of Interest

The authors declare that the research was conducted in the absence of any commercial or financial relationships that could be construed as a potential conflict of interest.

## Publisher's Note

All claims expressed in this article are solely those of the authors and do not necessarily represent those of their affiliated organizations, or those of the publisher, the editors and the reviewers. Any product that may be evaluated in this article, or claim that may be made by its manufacturer, is not guaranteed or endorsed by the publisher.

## Supplementary Material

Supplementary Figure 1 Expression patterns at the different development stages of three functional orthologs in the physical interval of *QSns.sau-AM-2B.2*. **(A)** *TRIDC2BG075290*; **(B)** *TRIDC2BG075310*; and **(C)** *TRIDC2BG075320*.

Supplementary Table 1 Environmental information of the surveyed agronomic traits in AM population.

Supplementary Table 2 The 1,150 bin markers were used for quantitative trait loci (QTLs) mapping.

Supplementary Table 3 Comparison of the genetic and physical positions of 6,537 mapped markers (including bin markers and co-segregated markers).

Supplementary Table 4 Epistasis interaction analysis of QTL for spikelet number per spike (SNS).

Supplementary Table 5 QTL detected in the QTL-by-environment interaction (QEI) module.

Supplementary Table 6 Details of kompetitive allele-specific PCR (KASP) markers.

Supplementary Table 7 Comparison of the major QTL with loci previously reported for SNS.

Supplementary Table 8 Predicted genes in the physical interval of *QSns.sau-AM-2B.2* and *QSns.sau-AM-3B.2*.
